# Exploring large-scale gene coexpression networks in peach (*Prunus persica* L.): a new tool for predicting gene function

**DOI:** 10.1093/hr/uhad294

**Published:** 2024-01-02

**Authors:** Felipe Pérez de los Cobos, Beatriz E García-Gómez, Luis Orduña-Rubio, Ignasi Batlle, Pere Arús, José Tomás Matus, Iban Eduardo

**Affiliations:** Institut de Recerca i Tecnologia Agroalimentàries (IRTA) , Mas Bové, Ctra. Reus-El Morell Km 3,8 43120 Constantí Tarragona, Spain; Centre de Recerca en Agrigenòmica (CRAG), Institut de Recerca i Tecnologia Agroalimentàries (IRTA), CSIC-IRTA-UAB-UB. Cerdanyola del Vallès (Bellaterra), 08193 Barcelona, Spain; Centre for Research in Agricultural Genomics (CRAG) CSIC-IRTA-UAB-UB, Campus UAB, Edifici CRAG, Cerdanyola del Vallès (Bellaterra), 08193 Barcelona, Spain; Centre de Recerca en Agrigenòmica (CRAG), Institut de Recerca i Tecnologia Agroalimentàries (IRTA), CSIC-IRTA-UAB-UB. Cerdanyola del Vallès (Bellaterra), 08193 Barcelona, Spain; Centre for Research in Agricultural Genomics (CRAG) CSIC-IRTA-UAB-UB, Campus UAB, Edifici CRAG, Cerdanyola del Vallès (Bellaterra), 08193 Barcelona, Spain; Institute for Integrative Systems Biology (I2SysBio), Universitat de Valencia-CSIC, Paterna, 46908, Valencia, Spain; Institut de Recerca i Tecnologia Agroalimentàries (IRTA) , Mas Bové, Ctra. Reus-El Morell Km 3,8 43120 Constantí Tarragona, Spain; Centre de Recerca en Agrigenòmica (CRAG), Institut de Recerca i Tecnologia Agroalimentàries (IRTA), CSIC-IRTA-UAB-UB. Cerdanyola del Vallès (Bellaterra), 08193 Barcelona, Spain; Centre for Research in Agricultural Genomics (CRAG) CSIC-IRTA-UAB-UB, Campus UAB, Edifici CRAG, Cerdanyola del Vallès (Bellaterra), 08193 Barcelona, Spain; Institute for Integrative Systems Biology (I2SysBio), Universitat de Valencia-CSIC, Paterna, 46908, Valencia, Spain; Centre de Recerca en Agrigenòmica (CRAG), Institut de Recerca i Tecnologia Agroalimentàries (IRTA), CSIC-IRTA-UAB-UB. Cerdanyola del Vallès (Bellaterra), 08193 Barcelona, Spain; Centre for Research in Agricultural Genomics (CRAG) CSIC-IRTA-UAB-UB, Campus UAB, Edifici CRAG, Cerdanyola del Vallès (Bellaterra), 08193 Barcelona, Spain

## Abstract

Peach is a model for *Prunus* genetics and genomics, however, identifying and validating genes associated to peach breeding traits is a complex task. A gene coexpression network (GCN) capable of capturing stable gene–gene relationships would help researchers overcome the intrinsic limitations of peach genetics and genomics approaches and outline future research opportunities. In this study, we created four GCNs from 604 Illumina RNA-Seq libraries. We evaluated the performance of every GCN in predicting functional annotations using an algorithm based on the ‘guilty-by-association’ principle. The GCN with the best performance was COO300, encompassing 21 956 genes. To validate its performance predicting gene function, we performed two case studies. In case study 1, we used two genes involved in fruit flesh softening: the endopolygalacturonases *PpPG21* and *PpPG22*. Genes coexpressing with both genes were extracted and referred to as melting flesh (MF) network. Finally, we performed an enrichment analysis of MF network and compared the results with the current knowledge regarding peach fruit softening. The MF network mostly included genes involved in cell wall expansion and remodeling, and with expressions triggered by ripening-related phytohormones, such as ethylene, auxin, and methyl jasmonate. In case study 2, we explored potential targets of the anthocyanin regulator PpMYB10.1 by comparing its gene-centered coexpression network with that of its grapevine orthologues, identifying a common regulatory network. These results validated COO300 as a powerful tool for peach and *Prunus* research. This network, renamed as PeachGCN v1.0, and the scripts required to perform a function prediction analysis are available at https://github.com/felipecobos/PeachGCN.

## Introduction

The advent of omics technologies has allowed the scientific community to generate enormous amounts of biological information. In parallel, increasingly efficient bioinformatic tools help us transform this information into structured biological knowledge. To date, more than seven million RNA-Seq libraries are available at the National Center of Biotechnology Information (NCBI, https://www.ncbi.nlm.nih.gov/), representing a great opportunity for large-scale bioinformatics exploration and biological data integration. Therefore, taking advantage of this valuable resource is essential in the age of big data analysis.

In transcriptomics, representing this complex data as gene coexpression networks (GCNs) is becoming a widespread practice. GCNs are usually represented as undirected graphs, where nodes correspond to genes and edges correspond to correlations in gene expression patterns. GCNs can be built across multiple experimental conditions (condition-independent GCNs) or in specific experimental conditions (condition-dependent GCNs, e.g. tissue specific GCNs). They are based on the ‘guilt-by-association’ (GBA) principle [1], which states that genes with related functions share similar expression patterns. Following this principle, and using the functional annotation of the genes within a network, GCNs can be a very powerful tool to infer gene functions and to understand the regulation of specific metabolic pathways. For this reason, GCNs are extremely useful in crop species, where most of the bioinformatic and genetic tools are modest and our understanding of gene function is still limited [2]. Several studies have already created GCNs in the plant model *Arabidopsis thaliana* ( [3–6]), maize ( [7]; S. [8]), rice [9, 10], wheat [5], and grapevine ( [11–14]).

Peach [*Prunus persica* L. (Batsch)] has been used as a model organism for genetics and genomics in the *Rosaceae*, and more specifically in the *Prunus* genus, which encompasses other crops such as sweet and tart cherry, European and Japanese plum, apricot, and almond. However, in peach, the validation of genes responsible for breeding traits is a complex task. Long intergeneration times, phenological cycles, and space constraints due to the large size of the individuals under study are some of the hindrances for the work of peach geneticists [15]. Moreover, there is a lack of efficient genetic transformation systems [16, 17]. As a result of these limitations, only two genes, *DRO1* and *TAC1*, have been biologically validated based on mutant analysis [18, 19].

Although small-scale condition-dependent GCNs have been reported in peach and other *Prunus* species [20–25], these were created *ad-hoc* to study specific biological processes and so cannot be used in other experimental contexts. Therefore, a GCN capable of capturing robust gene–gene relationships across various experimental conditions, developmental stages and tissues, is needed. A GCN with these characteristics would help researchers overcome the intrinsic limitations of peach genetic studies and outline future research opportunities.

In this study, we present the first large-scale GCN in peach. We constructed four GCNs from publicly available RNA-Seq data and evaluated the performance of every GCN using a machine-learning algorithm based on the GBA principle. The GCN with the best performance was validated by predicting gene functions of well-characterized genes. Finally, we provide the scripts and data needed for function prediction analyses using the GCN presented in this study. These resources can be found at https://github.com/felipecobos/PeachGCN.

## Results

### Aggregated GCNs included 82% of the protein-coding genes annotated in the peach reference genome

Six hundred and eight public RNA-Seq libraries, belonging to different organs and developmental stages of *P. persica,* were downloaded from SRA, classified and reanalyzed to generate different GCN types. To understand the differences between each GCN generated, we analyzed general topological characteristics of the four GCNs inferred in this study (Table 1). The two aggregated GCNs (COO100 and COO300) had 21 956 genes, while the other two, built by non-aggregated methods (HRR100 and HRR300) had 17 505 genes. From the total number of 26 873 protein-coding genes annotated in the peach reference genome, this represented 81.7% for aggregated and 65.1% for non-aggregated networks. The number of genes present in the aggregated GCNs represented 16.6% more genes (4451) from the peach whole-genome annotation, compared to non-aggregated GCNs.

**Table 1:** General topological characteristics of non-aggregated and aggregated GCNs with 100 and 300 top coexpressed genes (HRR100, HRR300, COO100, and COO300)

GCN	Number of genes	*P. persica* genes included in the GCN (%)	Range of node degree connectivity (min-max)	Average node degree connectivity
HRR100	17 505	65.1	649 (100–749)	161
HRR300	17 505	65.1	1490 (300–1790)	470
COO100	21 956	81.7	315 (100–415)	149
COO300	21 956	81.7	785 (300–1085)	442

**Figure 1:**
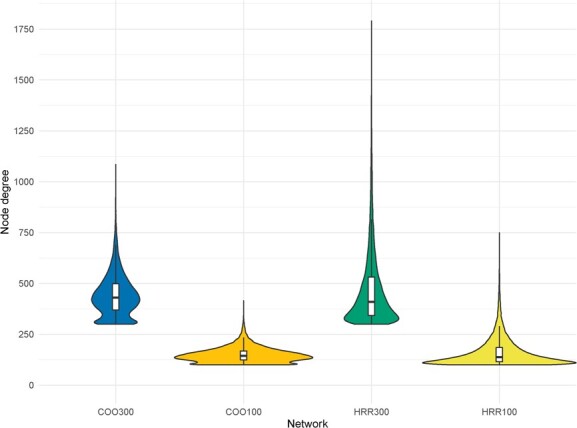
Violin plot of node degree connectivity in each of the aggregated and non-aggregated networks with relaxed or stringent sparsity (COO300, COO100, HRR300, and HRR100). Boxplots of node degree connectivity were added for each violin plot.

Node degree refers to the number of connections between nodes in a network. The different methods used not only affected the number of genes included in the network, but also the node degree connectivity across all nodes of the GCN. Average node degree connectivity was higher in networks with relaxed sparsity (442 in COO300 and 470 in HRR300) in comparison to stringent sparsity (149 in COO100 and 161 in HRR100). The range between minimum and maximum node degree connectivity is wider in non-aggregated GCNs compared to aggregated GCNs with the same sparsity threshold (comparing HRR300 with COO300 and HRR100 with COO100). The minimum node degree connectivity was set by the sparsity threshold in all the networks: 100 for stringent sparsity (HRR100 and COO100) and 300 for relaxed sparsity (HRR300 and COO300). The highest node degree connectivity was found in HRR300, with a maximum of 1790 coexpressed genes with one single gene. In addition, aggregated GCNs showed a bimodal node degree connectivity distribution while non-aggregated GCNs had a unimodal distribution (Figure 1).

### COO300 was the GCN with the highest AUROC value

When considering sparsity threshold, both GCNs with relaxed sparsity (HRR300 and COO300) had AUROC values above 0.7 for all the annotated databases (Table 2). COO300 was the GCN with the highest average AUROC value (0.746), outperforming the other GCNs. COO300 had the highest mean AUROC in almost all the datasets, except for Pfam and PANTHER, where the performance of HRR300 was better than that of COO300. HRR100 and COO100 had AUROC values under 0.7 in almost all the functional annotation databases, except for GOcc and PANTHER datasets. The best functional annotation performance in all the networks was for the functional annotation GOcc with an average AUROC value of 0.761, followed by PANTHER (0.724), KEGG (0.718), Pfam (0.714), GObp (0.709), Mapman (0.706), and GOmf (0.693).

**Table 2:** AUROC values for each GCN (COO300, HRR300, COO100, HRR100) performance in the different datasets. The best performance by dataset was highlighted with an asterisk


GCN	GObp	Gomf	GOcc	Pfam	KEGG	PANTHER	MapMan	Average
COO300	0.738*	0.723*	0.788*	0.736	0.750*	0.746	0.741*	0.746*
HRR300	0.724	0.705	0.773	0.745*	0.728	0.749*	0.732	0.736
COO100	0.681	0.670	0.733	0.680	0.697	0.688	0.664	0.687
HRR100	0.692	0.673	0.748	0.695	0.695	0.712	0.686	0.700
Average	0.709	0.693	0.761	0.714	0.718	0.724	0.706	0.717

The method used for network building also affected its performance, but the effect was not consistent. Considering the effect of the sparsity threshold, average AUROC values for relaxed sparsity threshold were always higher (HRR300 and COO300 = 0.741) than for the stringent threshold (HRR100 and COO100 = 0.694). When comparing GCNs by aggregation method, at relaxed sparsity (HRR300 and COO300), the average AUROC value for the aggregated method was higher but comparing at the stringent threshold (HRR100 and COO100), the average AUROC value was better for the non-aggregated method.

**Figure 2:**
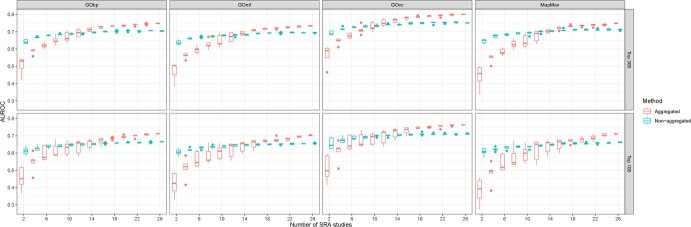
Boxplots of the AUROC value for every subset of Bioprojects (from 2 to 26) and method used.

Finally, we evaluated the effects of adding further experiments (i.e. Bioprojects) on the AUROC value of every GCN built in this study. Figure 2 shows the correlation between the network AUROC value and the number of Bioprojects used. For every combination of GCN building method (aggregated or non-aggregated), threshold (top 300 or top 100) and dataset used (GObp, GOmf, GOcc, and MapMan) we observed similar trends, where the AUROC value increased with the number of Bioprojects. This trend was more pronounced for aggregated GCNs than non-aggregated GCNs, reaching a plateau after adding 10 to 12 bioprojects. In all cases, the standard deviation of aggregated GCNs decreased as the number of Bioprojects increased.

### Aggregated GCNs showed a positive trend between average node degree connectivity and AUROC score of individual functional annotations for GObp, GOmf, GOcc, KEGG, and Mapman

**Figure 3:**
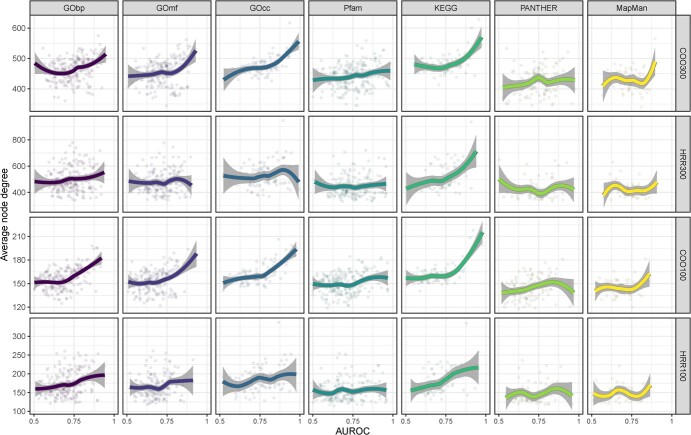
Scatter plot and Loess regression representation of average node degree connectivity by AUROC value for each of the GCNs (COO300, HRR300, COO100 and HRR100) in all the datasets used for network annotation (CObp, GOmf, GOcc, Pfam, KEEG, PANTHER and Mapman).

To assess the relationship between the AUROC score of individual functional annotations and the average node degree connectivity of the genes sharing that annotation we used a Loess regression (Figure 3). For example, for an individual functional annotation, such as *GOcc: cell wall*, we studied if the individual AUROC score of *GOcc: cell wall* was related to the average number of connections of the genes sharing that particular GO annotation. We then repeated the analysis for all the functional annotations within a dataset (*GOcc: apoplast*, *GOcc: extracellular region*, etc). In the case of aggregated GCNs, there was a positive trend between average node degree connectivity and AUROC score of individual functional annotations for GObp, GOmf, GOcc, KEGG, and Mapman. In the case of non-aggregated GCNs, the only dataset with a positive trend between average node degree connectivity and AUROC score of individual functional annotations was KEGG. The average node degree connectivity had no effect on the AUROC score of individual functional annotations in the Pfam dataset in any of the GCNs studied.

### Case study 1: The melting flesh network

We selected two well-characterized genes responsible for fruit flesh softening in peach, the endopolygalacturonases *PpPG21* and *PpPG22*, located on chromosome 4 (Table 3) [29–34]. Based on the evidence available to date, the variability of flesh softening and stone adhesion during fruit ripening is due to the allelic combination of these two homologous genes. Both genes, *PpPG21* or *PpPG22*, are associated with the development of melting, non-melting, or non-softening fruits, while *PpPG22* is also associated with the development of freestone or clingstone fruits.

**Table 3:** Candidate genes selected for network validation. The gene IDs were referred to the peach reference genome version 1 and 2.0 [26, 27] and NCBI [28] while genomic coordinates and annotation were referred to the peach reference genome version 2.0 [27]

Gene ID	Gene name	Genomic coordinates	Annotation
*Prupe.4G261900* *ppa006839m* *LOC18781156*	*PpPG21* *PpPG2* *PpPGM*	Chr04: 19046344–19 049 605 (+)	Involved in fruit ripening. Promotes flesh softening.
*Prupe.4G262200* *ppa006857m* *LOC18779267*	*PpPG22* *PpPG1* *PpPGF*	Chr04: 19081325–19 083 984 (+)	Involved in fruit ripening. Promotes flesh softening and stone detaching from mesocarp.
*Prupe.3G163100* *ppa026640m* *LOC18783018*	*PpMYB10* *PpMYB10.1*	Chr04: 18220455–18 222 943 (−)	Involved in fruit coloration. Promotes anthocyanin accumulation in fruit peel and flesh around the stone.

*PpPG21* and *PpPG22* gene-centered networks were functionally annotated using GObp, GOmf, GOcc, Pfam, KEGG, PANTHER, and MapMan term-classification schemes. Both networks shared several functional annotations: ‘*GOcc: extracellular region’*, ‘*GOcc: cell wall’*, ‘*GObp: metabolic process’*, ‘*GObp: cell wall organization’*, ‘*GOmf: hydrolase activity, acting on glycosyl bounds’*, ‘*GOmf: polygalacturonase activity’*, ‘*Mapman: enzyme classification. hydrolases. Glycoxylases’,* and ‘*Pfam: glycosyl hydrolases family 28’*. *PpPG22* was in turn exclusively associated with ‘*GObp: fruit ripening’* and ‘*GObp: carbohydrate metabolic process’* terms.

**Figure 4:**
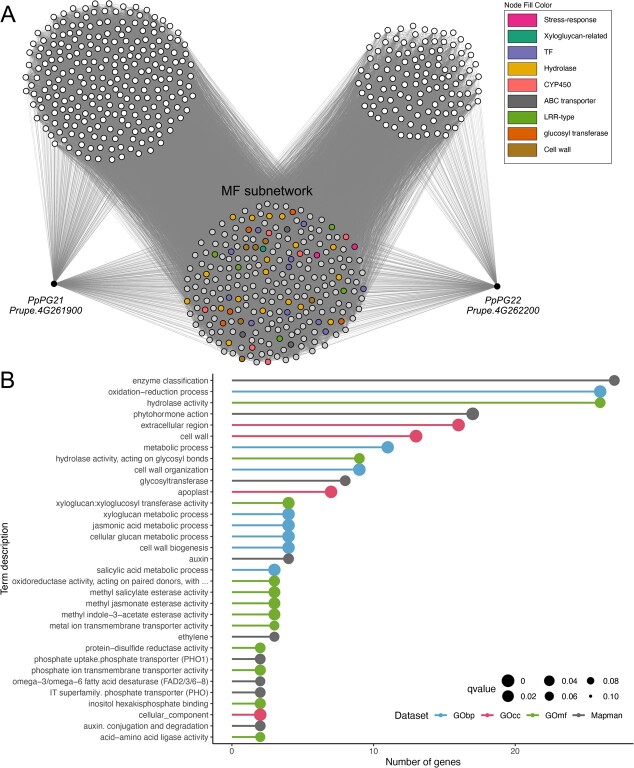
MF network analysis. (A) *PpPG21* and *PpPG22* GCNs and the MF network. Biological processes of interest are highlighted in the MF network. TF: transcription factor, CYP450: cytochrome P450. (B) Gene set enrichment analysis of the MF network.

The *PpPG21* and *PpPG22* gene-centered networks were constituted by 485 and 354 genes, respectively. Despite *PpPG21* and *PpPG22* were not found to be mutually coexpressed, their networks shared 238 genes. These shared genes were selected and named as the melting flesh (MF) network (Figure 4A; Supplementary Material S2). MF network was annotated in GObp, GOcc, GOmf, and Mapman datasets. From the 238 genes in the MF network, 136 genes showed significantly enriched terms in GObp, 123 in GOcc, 156 in GOmf and 116 in Mapman (Supplementary Material S2).

After MF network annotation, we performed an enrichment analysis. MF network was enriched in 33 different terms (33 terms were significantly over-represented in this subnetwork). Out of these 33 terms, 12 belonged to the GOmf dataset, 9 to Mapman, 8 to GObp, and 4 to GOcc (Figure 4B; Supplementary Material S2). Within GOmf terms, up to 26 genes were annotated as *hydrolase activity* or as its child term (direct descendant), *hydrolase activity, acting on glycosyl bonds*. The next term was ‘*xyloglucan:xyloglucosyl transferase activity’*, with four genes annotated. With three genes annotated, we found the terms ‘*methyl indole-3-acetate esterase activity’*, ‘*methyl salicylate esterase activity’*, ‘*methyl jasmonate esterase activity’*, ‘*oxidoreductase activity, acting on paired donors, with oxidation of a pair of donors resulting in the reduction of molecular oxygen to two molecules of water* and *metal ion transmembrane transporter activity’*. Finally, with two genes annotated, we found the terms ‘*inositol hexakisphosphate binding’*, ‘*phosphate ion transmembrane transporter activity’*, ‘*protein-disulfide reductase activity’*, and ‘*acid–amino acid ligase activity’*.

Using Mapman as the annotation dataset, 27 genes were annotated as ‘*enzyme classification’*. There were eight genes annotated as ‘*glycosyltransferase’*, a child term of ‘*enzyme classification’*. The next term, with 17 genes annotated, was ‘*phytohormone action’*. There were four genes annotated as ‘*auxin’* or ‘*auxin.conjugation and degradation’* and three as ‘*ethylene’*, child terms of ‘*phytohormone action’*. With two genes annotated, we found the terms ‘*Solute transport.carrier-mediated transport.IT superfamily.phosphate transporter (PHO)’*, ‘*Nutrient uptake.phosphorus assimilation.phosphate uptake.phosphate transporter (PHO1)’* and ‘*Lipid metabolism.fatty acid biosynthesis.fatty acid desaturation.omega-3/omega-6 fatty acid desaturase (FAD2/3/6-8)’*.

Within GObp, there were 26 genes annotated as ‘*oxidation–reduction process’*. Up to 11 genes were annotated as ‘*metabolic process’*. There were nine genes annotated as ‘*cell wall organization,’* and four as ‘*cell wall biogenesis’*, child terms of ‘*cell wall organization or biogenesis’*. There were four genes annotated as ‘*cellular glucan metabolic process’* and its child term, ‘*xyloglucan metabolic process’*, four as ’*jasmonic acid metabolic process’,* and three as ‘*salicylic acid metabolic process’*. Using GOcc as the annotation dataset, 16 genes were annotated as ‘*extracellular region’*, 7 genes as ‘*apoplast’*, child term of ‘*extracellular region’*, and up to 13 genes were annotated as ‘*cell wall’*.

We further explored the expression patterns of the MF network genes by inspecting all the SRA runs used to create the whole-genome networks. Those runs with enough metadata to be classified in organs were kept and used to calculate normalized expression values. Most MF-genes had specific or at least preferential expression in fruit tissues (Supplementary Figure S2).

### Case study 2: The fruit color network

**Figure 5:**
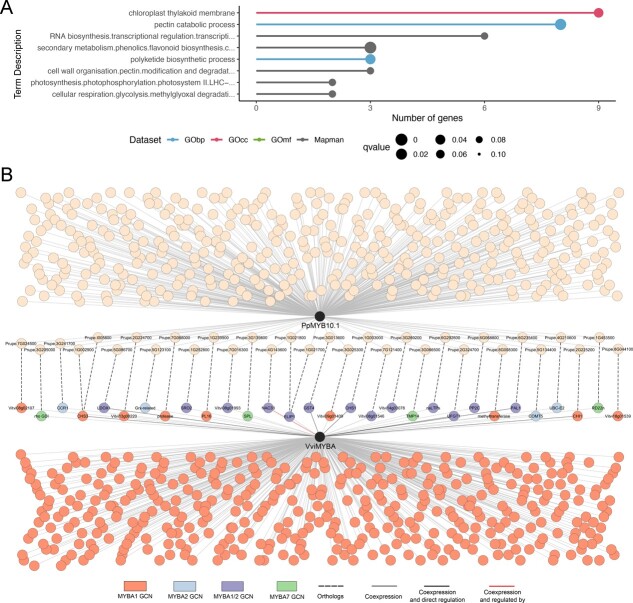
FC network analysis. (A) Gene set enrichment analysis of the FC network. (B) Dual species comparison of FC network and *VviMYBA1*/*VviMYBA2* gene centered networks. For details see Supplementary Material S3.

We selected the transcription factor *PpMYB10.1*, a subgroup 6 R2R3-MYB member, responsible of controlling anthocyanin accumulation in peach fruit tissues, to create the fruit color (FC) network ( [35–43]). *PpMYB10.1* gene-centered network was constituted by 419 genes. This FC network was functionally annotated using the GObp, GOcc, GOmf, and Mapman classifications. Out of the 419 genes in the FC network, 227 genes were annotated in GObp, 207 in GOcc, 246 in GOmf and 192 in Mapman (Supplementary Material S2). The enrichment analysis of the FC network resulted in eight enriched terms. Five belonged to Mapman dataset, two to GObp, and one to GOcc. No enriched term belonged to GOmf dataset (Figure 5A; Supplementary Material S2). Within Mapman, six genes were annotated as ‘*RNA biosynthesis.transcriptional regulation. transcription factor (C2H2-ZF)*’. Three genes were annotated with the terms ‘*secondary metabolism.phenolics.flavonoid biosynthesis.chalcones.chalcone synthase activity.chalcone synthase (CHS)*’ and ‘*cell wall organization.pectin.modification and degradation.pectate lyase*’. Finally, two genes were annotated with the terms ‘*photosynthesis.photophosphorylation. photosystem II.LHC-related protein groups.three helix LHC-related protein group.protein (ELIP)*’ and ‘*cellular respiration.glycolysis.methylglyoxal. degradation.glutathione-independent glyoxalase (GLY-III)*’. Using GObp as the annotation dataset, there were eight genes annotated as ‘*pectin catabolic process*’ and three genes annotated as ‘*polyketide biosynthetic process*’. Finally, using GOcc as annotation dataset, there were nine genes annotated as ‘*chloroplast thylakoid membrane*’.

Since PpMYB10.1 is a transcription factor, we explored its GCN with the idea to identify potential targets. The color-related PpMYB10.1 network was compared with tissue-independent and berry-dependent gene-centered networks of its grapevine orthologs VviMYBA1/VviMYBA2 and VviMYBA7, responsible for the anthocyanin pigmentation of fruits (Walker et al., 2007) and stress-induced pigmentation of vegetative organs (Matus et al., 2017), respectively. The comparison of both gene-centered networks show 10% of shared co-expressed orthologs (Figure 5B), from which many are known as direct targets of grapevine MYBA regulators, such as the glycosyl-transferase-coding gene Vvi*UFGT1*, the glutathione-S-transferase Vvi*GST4* and chalcone synthase Vvi*CHS3*, among others. *VviNAC33* is known as a regulator of Subgroup 6 MYBs in grape (D'Incà et al., 2023) and both peach and Vitis networks show co-expression of the NAC ortholog with *PpMYB10* and *VviMYBA* genes, respectively. The peach FC network shows differences in conserved coexpression depending on the *MYBA* genes considered. The biggest similarity is found with fruit-specific Vvi*MYBA1* and Vvi*MYBA2* genes.

## Discussion

### Different GCN topological features are affected by the selected algorithms

To achieve the best results when building gene coexpression networks (GCNs), two variables were tuned, aggregation method and sparsity threshold. We chose these variables since they are the ones that have the greatest influence on the subsequent performance of the GCNs [13]. The four GCNs obtained were evaluated, with substantial differences in the general topological characteristics of the inferred GCNs.

When considering GCN building methods, a major difference between aggregated and non-aggregated GCNs was the number of genes constituting the network. Aggregated GCNs had 21 956 genes (81.7% of *P. persica* genes), while non-aggregated GCNs only had 17 505 (65.1% of *P. persica* genes). This difference comes from the low-expression gene filtering. In non-aggregated GCNs all the genes with less than 0.5 FPKM in 50% of the 498 RNA-Seq libraries were filtered, while in aggregated GCNs this filtering is independently performed for each of the 26 Bioproject groups. This allowed the inclusion in the GCN of genes expressed in more precise conditions and therefore involved in more specific processes. This indicates that both aggregated and non-aggregated networks are able to capture stable gene–gene relationships expressed in most of the RNA-Seq libraries used in the analysis, but only aggregated GCNs are able to detect gene–gene interactions produced in specific conditions. Condition-independent gene–gene connections could be related to basal metabolic pathways, while condition-dependent gene–gene interactions could be associated to specific metabolic pathways. This could explain the difference in the distribution of node degree connectivity between aggregated and non-aggregated GCNs. In scale-free topology networks (most cases in biology-related networks), degree is not distributed homogeneously across nodes; instead, some nodes may have a very high degree, highlighting them as putative network hubs. This property is what actually makes GCNs a suitable tool to precit gene function. As shown in Figure 1, aggregated GCNs had a bimodal distribution of node degree connectivity (it had two peaks), while non-aggregated GCNs had a unimodal distribution (only one peak). As previously mentioned, aggregated GCNs may be able to detect genes involved in specific and basal metabolic processes. The two modes detected in aggregated GCNs node degree connectivity distribution could be associated with these two groups of genes. The group with the lower node degree distribution could be associated with genes involved in more specific metabolic pathways, coexpressed with a lower number of genes. The group with the higher node degree distribution could be associated with genes involved in basal metabolic pathways and coexpressed with a higher number of genes. On the other hand, non-aggregated GCNs may only detect genes involved in basal metabolic pathways, having only one mode in their node degree distribution.

Another factor affecting the topology of the networks was the sparsity threshold selected. HRR300 and COO300 had a node degree connectivity higher than HRR100 and COO100. This was an expected result, since a higher number of ranked genes allows a higher number of connections between genes.

### Sparsity threshold and the number of bioprojects determine network performance

According to the results, sparsity was a key factor affecting network performance. The average AUROC of relaxed sparsity threshold networks (HRR300 and COO300) was 0.741, while that of stringent sparsity threshold networks (HRR100 and COO100) was 0.694. Applying relaxed sparsity threshold during network building represented an increment of 6.3% in the AUROC score in comparison to stringent sparsity threshold.

The number of bioprojects used to build the GCN was a key factor in the case of aggregated methods, indicating the minimum number of Bioprojects necessary to reach a sufficiently high AUROC score (Figure 1). In every case, aggregated methods had a lower AUROC value than non-aggregated methods using a low number of Bioprojects. By increasing this number, aggregated methods overtook non-aggregated methods, as found in other studies [11, 12]. For future GCNs construction, increasing the number of Bioprojects could improve the overall performance of the GCNs.

While studying the effect of functional annotations average node degree on the AUROC value, we found major differences depending on the type of used dataset. There was a positive correlation between functional annotations average node degree and functional annotations individual AUROC in evidence-based datasets such as GObp, GOmf, GOcc, KEGG, and Mapman. On the other hand, this correlation was lost with datasets based on domain identification by sequence similarity, such as PANTHER and Pfam. These results are in agreement with the GBA principle, which states that coexpressed genes share function, and not necessarily similar sequences.

### COO300 validated as a powerful tool for peach and *Prunus* research

In peach, fruit flesh softening has been extensively studied at fruit ripening and postharvest due to its implication in fruit shelf life. Fruit softening involves several cellular processes, such as the disassembly of the cell wall and the dissolution of the middle lamella. These modifications are the result of hydrolytic changes in the polysaccharides that form the cell wall, including celluloses, hemicelluloses (mainly xyloglucan) and pectins. In case study 1, several terms found in the melting flesh (MF) network were associated to this process, such as ‘*GOcc: cell wall’*, ‘*GObp: cell wall organization’,* and ‘*GObp: cell wall biogenesis’*, ‘*GOmf: hydrolase activity’*, ‘*GOmf: hydrolase activity, actin on glycosyl bonds’*, ‘*Mapman: enzyme classification.EC_2 transferases.EC_2.4 glycosyltransferase’,* and ‘*GOmf: xyloglucan:xyloglucosyl transferase activity’*.

Peach flesh softening is a synergistic process triggered by an extensive phytohormone signaling network. As a climacteric fruit, cross talk between ethylene and auxin occurs during peach ripening [44]. Moreover, methyl jasmonates (MeJAs) play an important role in slowing down fruit ripening by inhibiting ethylene production and fruit flesh softening [45, 46]. Up to seven enriched terms were related to these phytohormones in the MF network, such as ‘*Mapman: phytohormone action’*, ‘*Mapman: phytohormone action. Auxin’*, ‘*GObp: jasmonic acid metabolic process’*, ‘*Mapman: phytohormone action. ethylene’, ‘GOmf: methyl indole-3-esterase activity’*, ‘*GOmf: methyl jasmonate esterase activity’,* and ‘*Mapman: phytohormone action. auxin. auxin conjugation and degradation’*.

We found 25 genes in the MF network that have previously been reported as associated to ripening and softening (Supplementary Material S2). Among them, we identified several genes involved in the enzymatic machinery responsible for cell wall disassembly, such as a pectin methylesterase (*Prupe.7G192800*), a pectin methylesterase inhibitor (*Prupe.1G114500*), a pectate lyase (*Prupe.4G116600*), a β-galactosidase (*Prupe.3G050200*), and a xyloglucan endotransglycosylase hydrolase (*Prupe.1G255100*). Additionally, we found an expansin, a cell wall structural protein (*Prupe.6G075100*). Related to ethylene, we identified a 1-amino-cyclopropane-1-carboxylate synthase (*PpACS1*, *Prupe.2G176900*) and 1-amino-cyclopropane-1-carboxylate oxidase (*PpACO1*, *Prupe.3G209900*), both genes codifying the key enzymes catalyzing the final steps of the ethylene biosynthetic pathway [47]. In fact, *PpACS1* has been previously reported as a regulator of *PpPG21* [48]. Another gene related to ethylene production was the ethylene receptor 2 (*PpETR2*, *Prupe.1G034300*). The implication of this gene in the ethylene transduction signal has been verified at the transcriptional level in the final stages of fruit ripening in melting flesh peaches [49]*.* Regarding genes related to auxin biosynthesis, we found a YUCCA-like auxin-biosynthesis gene (*PpYUC11*, *Prupe.6G157500*) and an IAA amino acid synthase (*PpGH3*, *Prupe.6G226100*). Both genes have been reported to have the same expression pattern as *PpACS1* at late ripening stages in response to high auxins levels in melting flesh fruits [50].

Based on these results, we can affirm that the MF network is mainly constituted by genes involved in cell wall organization and biogenesis, with expression regulated by ripening-related phytohormones, such as ethylene, auxin, and methyl jasmonate. Moreover, we found 25 genes previously reported as involved in softening, some taking part in key steps of these processes. These results demonstrate that the MF network is closely related to peach fruit softening and therefore to the function of *PpPG21* and *PpPG22*.

In case study 2, we prospected the fruit color (FC) network, related to the transcriptional regulation of anthocyanin accumulation in peach fruit tissues (i.e. mesocarp and exocarp). Anthocyanins belong to the flavonoid compounds and are water-soluble pigments that determine the red-to-blue color of plant tissues in many species as peach [51, 52]. Their biosynthesis starts through the phenylpropanoid pathway in the cytosol, and then they are modified and transported to the vacuoles, where they finally accumulate. Anthocyanin spatial and temporal distribution is determined by the expression pattern of structural and regulatory genes. Furthermore, anthocyanin biosynthesis is controlled by environmental factors, such as light incidence in fruits and temperature in leaves [53–56]. One of the key genes is *PpMYB10.1*, that has been proposed as a strong candidate for the red pigmentation of the flesh around the stone and the red skin of the fruit [35–37, 39–43, 57].

The *PpMYB10.1*-gene centered network, called here FC network, was enriched with many terms directly involved in anthocyanin metabolism, such as *‘Mapman: secondary metabolism. phenolics. Flavonoid biosynthesis. chalcones. chalcone synthase activity. chalcone synthase (CHS)*’ and ‘*Obp: polyketide biosynthetic process’*. This and other related terms contain many genes that are orthologs of well-known anthocyanin-related genes in grapevine (*e.g.* the CHS gene *Prupe.1G002900, Prupe.1G003000* and *Prupe.I005800*). In fact, our Prunus-Vitis dual-species comparison allowed us to identify putative targets of PpMYB10 that can be subject of further molecular characterization. For instance, one of the *CHS* genes reported here is known to be directly regulated by the MYB10.1/bHLH3 complex [39].

Among the enriched terms related with anthocyanins metabolism we found two genes annotated as protein DJ-1 like (*Prupe.3G012200* and *Prupe.3G012800*), highly correlated with the transporter glutathione S-transferase 1 (PpGST1) (*Prupe.3G013600*) that is essential for vacuole sequestration of anthocyanins in peach [36, 42, 58]. Additionally, we found MYB10.3 (*Prupe.3G163300*), that is an orthologous of MYB10.1, phenylalanine ammonia-lyase (PAL) (*Prupe.6G235400*), 4-coumarate CoA ligase (4CL) (*Prupe.2G326300*), chalcone synthase (CHS) (*Prupe.1G002900* and *Prupe.1G003000*), chalcone isomerase (CHI) (*Prupe.2G225200*), dihydroflavonol reductase (DFR) (*Prupe.1G376400, Prupe.3G241700* and *Prupe.4G200500*), anthocyanidin synthase (ANS) (*Prupe.5G086700*), and UDP flavonoid 3-O-glucosyltransferase (UFGT) (*Prupe.2G324700* and *Prupe.8G131000*). It has been reported that the overexpression of MYB10.1/bHLH3 and MYB10.3/bHLH3 activated anthocyanin biosynthesis by up-regulating the anthocyanin biosynthetic genes chalcone synthase (CHS), dihydroflavonol reductase (DFR), and UDP flavonoid 3-O-glucosyltransferase (UFGT). Their expression was validated at the transcriptional level in peach fruit [36, 41, 43, 56, 59] and tobacco leaves [41, 43].

Among transcription factors, we found the gene transcription-related term ‘*Mapman*: *RNA biosynthesis. transcriptional regulation. transcription factor (C2H2-ZF)*’ enriched. A set of enriched genes were annotated as ‘*C2H2-type zinc finger transcription factors zinc finger of A. thaliana (ZAT) proteins*’ (*Prupe.2G230800, Prupe.5G068600, Prupe.5G201400, Prupe.6G084100, Prupe.6G249300* and *Prupe.7G151500*). This protein has been proposed as a regulator of anthocyanin biosynthesis under stress conditions in apple [60] and pear [61]. Additionally, a putative regulator of *MYB* expression was found while comparing the Prunus and Vitis networks; *Prupe.4G143600*, the ortholog of a NAC TF known to bind and activate Vvi*MYBA1*’s promoter region (ViNAC33; D'Inca et al., 2023).

Additional terms were related to regulation by environmental effects ‘*Mapman*: *photosynthesis.photophosphorylation. photosystem II.LHC-related protein groups.three helix LHC-related protein group.protein (ELIP)*’ and ‘*GOcc*: *chloroplast thylakoid membrane’*, also shared in MYBA GCNs genes related with the thylakoid membranes conforming the photosystem II in interaction with light-harvesting complex (PSII–LHCII) acting as light-harvesting supercomplexes [62] (*Prupe.1G021700*, *Prupe.1G021800. Prupe.6G259200, Prupe.2G104500*, *Prupe.3G066500,* and *Prupe.6G173300*). Many of the orthologous genes found in Arabidopsis codify for the family of light-harvesting-related proteins as Early Light-Induced Proteins (ELIP1, AT3G22840.1; ELIP2, AT4G14690.1), LHCs (Stress Enhanced Protein 2, SEP2, AT2G21970.1), and Hight Light-Induced Proteins/One Helix Protein (HLIP/OHP, AT5G02120.1). Although they could be related with the induction of anthocyanins by light, its implication in high light- and cold-stress responses and photooxidative protection may lead their main function in peach fruit [63].

Finally, a set of annotated terms related to cell wall organization was found in the FC network, probably due to the temporal and spatial co-localization of anthocyanin biosynthesis and pectin degradation during fruit ripening ‘*Mapman*: *cell wall organization. pectin. modification and degradation. pectate lyase*’ and ‘*GObp*: *pectin catabolic process*’. These genes are involved in pectin modification during cell wall organization and include pectinesterase inhibitors (PEI) (*Prupe.1G131900* and *Prupe.1G123800*), pectate lyase (PL) (*Prupe.1G239900, Prupe.1G268500* and *Prupe.1G268700*), a polygalacturonase (*Prupe.2G014800*), and two pectin methylesterases (PME) (*Prupe.3G003700* and *Prupe.3G147000*). Anthocyanins and cell wall degradation enzymes have overlapping expression patterns in peach fruit at ripening stage, as already observed at the transcriptional level in the mesocarp and exocarp under UV-B radiation [54]. The gene pectate lyase 6 (PL6) is in fact also co-expressed to S6-MYBs in grapevine, representing either a potential MYB target or suggesting a common regulator with these type of MYB genes.

Taken the results obtained in case studies 1 and 2 together, we can affirm that COO300 is validated as an accurate and powerful tool for gene function prediction in *Prunus* sp.

### Gene coexpression networks as catalysts for *Prunus* research

While large-scale GCNs have not been explored yet as gene function-prediction tools in *Prunus* research, they have been widely used in the model organism *A. thaliana* and other crop species, such as grapevine. Depending on the needs of the researcher, GCNs can be exploited in different ways. One of the most common is to identify different modules (also known as clusters or hubs) within the GCN through a clusterization analysis. These gene modules, which represent groups of genes highly connected between them and relatively isolated from the rest of the GCN, are particularly useful to study uncharacterized biological processes. For example, [9] used this approach in rice to annotate 13 537 genes, from which 2980 had no previous annotation.

Another approach that uses group of genes to study specific biological processes is the gene group-guided analysis. In this case, a list of well-characterized genes involved in a specific biological process are selected and genes coexpressing with the list of genes of interest are extracted from the network. In this way, the selected genes are used as a guide to study the transcriptional regulation of the biological process of interest. Huang et al. [7] successfully applied this approach to study the cell wall biosynthesis in maize. Pathway-centered network analysis has also been helpful in the identification of members or regulators of secondary metabolic pathways [12].

Finally, GCNs can be used to infer the function of a gene of interest by extracting gene-centered GCNs. These can also be used to study specific gene families, being particularly useful for studying transcription factor families. For instance, [14] developed R2R3 MYB-centered GCNs to study the potential secondary metabolic processes regulated by this family in grapevine. Gene-centered GCNs are of special interest in peach and *Prunus* research, where most trait-loci analyses lead to a list of candidate genes associated with the trait under study. With poor or no functional information, identifying the responsible gene from this list of candidates can be almost impossible. Even when a high-confidence candidate gene is identified, the lack of an efficient genetic transformation system is still one of the main limitations for functional, mutant, or transgenic based validation. Having a tool, such as the GCN presented in this study, with which obtaining useful information about the biological processes in which a gene is involved, may be of critical importance.

## Conclusions

In this study, we performed the widest overview of transcriptomic analysis carried out to date in peach or other *Prunus* species. The GCN inference methods used, aggregated or non-aggregated, affected the topological characteristics and performance of the GCNs created. Using three well-characterized genes in peach, *PpPG21, PpPG22,* and *PpMYB10*, we were able to validate the network with the best performance, COO300. The GCN tool presented in this study will help *Prunus* researchers overcome the intrinsic limitations of working with crop tree species, prioritize research lines and outline new ones. COO300, named as PeachGCN v1.0, and the scripts necessary to run a function prediction analysis using it, are available at https://github.com/felipecobos/PeachGCN.

## Materials and methods

### Data compilation

Forty-nine independent Sequence Read Archive (SRA) Bioprojects, encompassing 608 RNA-Seq libraries (Supplementary Material S1) were downloaded from the SRA database [64] in the NCBI [28]. These RNA-Seq libraries represented all the libraries available in the NCBI to date 09/04/2020. The peach reference genome ‘Lovell’ version 2.1 [26, 27] and its functional annotation were downloaded from Genome Database for Rosaceae (GDR) [65]. Finally, seven functional gene annotation datasets were retrieved using the methods described below. Gene ontology peach functional terms for biological process (GObp), molecular function (GOmf), and cellular component (GOcc) [66, 67] and Pfam database peach classification [68] were retrieved using the biomaRt R package [69]. Kyoto Encyclopedia of Genes and Genomes (KEGG) peach pathway annotations [70] were retrieved using the KEGG API (https://www.kegg.jp/kegg/rest/keggapi.html). PANTHER HMM peach classifications version 16 [71] and MapMan Pathways version 4.2 [72] were downloaded from the public repositories.

### Mapping and quality filtering

We performed a sequencing-quality filtering and adapter removal using Trim Galore! version 0.6.1 (https://www.bioinformatics.babraham.ac.uk/projects/trim_galore/). Reads with terminal Ns were trimmed, then reads with a Phred score lower than 28 or smaller than 35 nucleotides were filtered. Filtered libraries were quality checked using FastQC version 0.11.5 (http://www.bioinformatics.babraham.ac.uk/projects/fastqc/). HISAT2 version 2.1 [73] was used to map RNA sequencing libraries to the reference peach genome ‘Lovell’ version 2.1 [26, 27] with default parameters. Mapped Binary Alignment Map (BAM) files were filtered by alignment quality using SAMtools version 1.9 [74, 75]. Reads with mapping quality lower than 40 were filtered out. After this filtering, BAM files with less than 5 000 000 reads were discarded, leaving a total of 498 RNA-Seq libraries from 43 independent Bioprojects for further analyses.

### Aggregated and non-aggregated GCNs inference

A raw count matrix was calculated using featureCounts [76], from Subread R package version 2.0.0 (http://subread.sourceforge.net/). For the raw count matrix construction, we excluded chimeric fragments and we used the coding DNA sequences as feature type and gene IDs as attribute type. The raw count matrix was then normalized to fragments per kilobase million (FPKM) mapped fragments (Z. [77]), obtaining a FPKM matrix. We then applied two different methodologies: aggregated and non-aggregated network inference with two sparsity thresholds set at top 100 (stringent threshold) and 300 (relaxed threshold) ranked genes (Supplementary Figure S1).

For non-aggregated analysis, genes with less than 0.5 FPKM in 50% of the RNA-Seq libraries were removed. Pearson's correlation coefficient (PCC) was calculated for the remaining genes and ranked according to descending PCC, giving a PCC matrix. High reciprocal rank networks for the top 100 (HRR100) and top 300 (HRR300) were constructed according to the formula:

$$ HRR\ \left(x,y\right)=\left[\mathit{\max}\left(\mathit{\operatorname{rank}}\left(x,y\right),\mathit{\operatorname{rank}}\left(y,x\right)\right)\right] $$

whereby *rank(x, y)* is the descending sorted rank of gene *y* according to the coexpression list of gene *x* and vice versa for *rank(y, x)*.

For aggregated analysis, we clustered the samples into 43 different groups according to the Bioproject study ID. We filtered Bioprojects with less than six RNA-Seq libraries, leaving 26 different groups with a total of 450 RNA-Seq libraries. Genes with less than 0.5 FPKM in 50% of the libraries within each group were removed and from each filtered FPKM matrix, a high reciprocal rank network for the top 100 and top 300 was constructed. Frequency of gene coexpression interactions in all groups was calculated and ranked in a co-occurrence matrix. Finally, co-occurrence networks for top 100 (COO100) and top 300 (COO300) interactions were obtained.

### Networks performance assay

Networks were evaluated for their ability to connect peach genes sharing functional annotations. For this purpose, GBA neighbor voting, a machine learning algorithm based on the GBA principle [78], was assessed over the GObp, GOmf, GOcc, Pfam, KEGG, PANTHER, and MapMan datasets. Each network was scored by the area under the receiver operator characteristic curve (AUROC) across all functional categories annotated for the seven datasets. Annotations were limited to groups containing 20 to 1000 genes to ensure robustness and stable performance when using neighbor voting. The AUROC value threshold for an acceptable network functional annotation was set at 0.7.

We also evaluated the impact of adding individual Bioprojects to the different networks created, HRR300, HRR100, COO300, and COO100. For this purpose, we selected five subsets each of two Bioprojects computing the top 100 and top 300 HRR and COO GCNs, evaluating their AUROC using GObp, GOmf, GOcc, and MapMan datasets. We repeated this process adding one Bioproject to the initial subset to reach five subsets each with 26 Bioprojects, the maximum number of Bioprojects used in this study. The final subsets corresponded to the full HRR300, HRR100, COO300, and COO100.

### Network validation

To validate the performance of COO300 in predicting gene functional annotations, we performed two case studies. In Case Study 1, we selected two well-characterized genes responsible for fruit flesh softening in peach, the endopolygalacturonases *PpPG21* and *PpPG22*, located on chromosome 4 (Table 3) [29–34]. Based on the evidence available to date, the variability of flesh softening and stone adhesion during fruit ripening is due to the allelic combination of these two homologous genes. Both genes, *PpPG21* or *PpPG22*, are associated with the development of melting, non-melting or non-softening fruits, while *PpPG22* is associated with the development of freestone or clingstone fruits.

To validate the performance of COO300 in predicting gene functional annotations, we performed two case studies (Table 3). As part of Case Study 1, genes coexpressed with *PpPG21* and *PpPG22* were extracted. Since both genes are involved in the peach fruit flesh softening process, we selected genes present in both subnetworks. The selected subnetwork was named melting flesh (MF) subnetwork.

In Case Study 2, we selected the transcription factor *PpMYB10.1*, responsible of anthocyanin accumulation in peach fruit ( [35–43]). The *PpMYB10.1* network, here named as fruit color (FC) network, was compared with *VviMYBA1*, *VviMYBA2* and *VviMYBA3* tissue independent and berry-dependent networks. The top 1% coexpressed genes (420 genes) were extracted from the AggGCNs app found in the Vitviz platform (http://vitviz.tomsbiolab.com/; [12]). Detection of peach-grapevine orthologues was conducted with Orthofinder (Emms & Kelly, 2019) using default parameters. As input datasets we used the *P. persica* proteome fasta file found in the Genome Database for Rosaceae (GDR, https://www.rosaceae.org/species/prunus_persica/genome_v2.0.a1) and the VCOST.v3 *Vitis vinifera* proteome from the 12X.2 assembly (Canaguier et al., 2017). Grapevine GCNs and orthology results are found in Supplementary Material S3.

MF and FC networks were functionally analyzed with enrichment analyses using GObp, GOmf, GOcc and Mapman classifications. Functional annotations statistically over-represented were selected if passed the significance threshold of *q*-value <0.1. Finally, we compared the enriched terms (the functional annotations statistically over-represented) of MF and FC networks with the current knowledge on the peach fruit softening process and skin anthocyanin accumulation, respectively.

## Supplementary Material

Web_Material_uhad294

## Data Availability

The PeachGCN v1.0, and the scripts necessary to run a function prediction analysis using it, are available at https://github.com/felipecobos/PeachGCN.

## References

[1] Oliver S . Guilt-by-association goes global. Nat Cell Biol. 2000;403:601–2 10.1038/35001165 10688178

[2] Schaefer RJ , Michno JM, Myers CL. Unraveling gene function in agricultural species using gene co-expression networks. Biochimica et Biophysica Acta (BBA) - Gene Regulatory Mechanisms. 2017;1860:53–63 27485388 10.1016/j.bbagrm.2016.07.016

[3] Amrine KCH , Blanco-Ulate B, Cantu D. Discovery of Core biotic stress responsive genes in Arabidopsis by weighted gene co-expression network analysis. PLoS One. 2015;10:e0118731. 25730421 10.1371/journal.pone.0118731 PMC4346582

[4] Furuya T , Saito M, Uchimura H. et al. Gene co-expression network analysis identifies BEH3 as a stabilizer of secondary vascular development in Arabidopsis. Plant Cell. 2021;33:2618–36 34059919 10.1093/plcell/koab151 PMC8408481

[5] Lv L , Zhang W, Sun L. et al. Gene co-expression network analysis to identify critical modules and candidate genes of drought-resistance in wheat. PLoS One. 2020;15:e0236186 32866164 10.1371/journal.pone.0236186 PMC7458298

[6] Mao L , Van Hemert JL, Dash S. et al. Arabidopsis gene co-expression network and its functional modules. Bioinformatics. 2009;10:1–24 10.1186/1471-2105-10-346 PMC2772859 19845953

[7] Huang J , Vendramin S, Shi L. et al. Construction and optimization of a large gene coexpression network in maize using RNA-seq data. Plant Physiol. 2017;175:568–83 28768814 10.1104/pp.17.00825 PMC5580776

[8] Ma S , Ding Z, Li P. Maize network analysis revealed gene modules involved in development, nutrients utilization, metabolism, and stress response. BMC Plant Biol. 2017;17:131–17 28764653 10.1186/s12870-017-1077-4 PMC5540570

[9] Childs KL , Davidson RM, Buell CR. Gene Coexpression network analysis as a source of functional annotation for Rice genes. PLoS One. 2011;6:e22196 21799793 10.1371/journal.pone.0022196 PMC3142134

[10] Ficklin SP , Luo F, Feltus FA. The Association of Multiple Interacting Genes with specific phenotypes in Rice using gene Coexpression networks. Plant Physiol. 2010;154:13–24 20668062 10.1104/pp.110.159459 PMC2938148

[11] Orduña L , Li M, Navarro-Payá D. et al. Direct regulation of shikimate, early phenylpropanoid, and stilbenoid pathways by subgroup 2 R2R3-MYBs in grapevine. Plant J. 2022;110:529–47 35092714 10.1111/tpj.15686

[12] Orduña-Rubio L , Santiago A, Navarro-Payá D. et al. Aggregated gene co-expression networks for predicting transcription factor regulatory landscapes in a non-model plant species. BioRxiv. 2023 10.1093/jxb/erad344 37668374

[13] Wong DCJ . Network aggregation improves gene function prediction of grapevine gene co-expression networks. Plant Mol Biol. 2020;103:425–41 32266646 10.1007/s11103-020-01001-2

[14] Wong DCJ , Schlechter R, Vannozzi A. et al. A systems-oriented analysis of the grapevine R2R3-MYB transcription factor family uncovers new insights into the regulation of stilbene accumulation. DNA Res. 2016;23:451–66 27407139 10.1093/dnares/dsw028 PMC5066171

[15] Aranzana MJ , Decroocq V, Dirlewanger E. et al. Prunus genetics and applications after de novo genome sequencing: achievements and prospects. 2019;6:58. 10.1038/s41438-019-0140-8 PMC6450939 30962943

[16] Limera C , Sabbadini S, Sweet JB. et al. New biotechnological tools for the genetic improvement of major Woody fruit species. Front Plant Sci. 2017;8:1418 28861099 10.3389/fpls.2017.01418 PMC5559511

[17] Ricci A , Sabbadini S, Prieto H. et al. Genetic transformation in peach (Prunus persica L.): challenges and ways forward. Plan Theory. 2020;9:971 10.3390/plants9080971 PMC7465125 32752031

[18] Dardick C , Callahan A, Horn R. et al. PpeTAC1 promotes the horizontal growth of branches in peach trees and is a member of a functionally conserved gene family found in diverse plants species. Plant J. 2013;75:618–30 23663106 10.1111/tpj.12234

[19] Guseman JM , Webb K, Srinivasan C. et al. DRO1 influences root system architecture in Arabidopsis and Prunus species. Plant J. 2017;89:1093–105 28029738 10.1111/tpj.13470

[20] García-Gómez BE , Ruiz D, Salazar JA. et al. Analysis of metabolites and gene expression changes relative to apricot (Prunus armeniaca L.) fruit quality during development and ripening. Front Plant Sci. 2020;11:1269 32973833 10.3389/fpls.2020.01269 PMC7466674

[21] Jiang X , Liu K, Peng H. et al. Comparative network analysis reveals the dynamics of organic acid diversity during fruit ripening in peach (Prunus persica L. Batsch). BMC Plant Biol. 2023;23:1–14 36617558 10.1186/s12870-023-04037-w PMC9827700

[22] Wang Q , Cao K, Li Y. et al. Identification of co-expressed networks and key genes associated with organic acid in peach fruit. Sci Hortic. 2023;307:111496

[23] Wu X , Du A, Zhang S. et al. Regulation of growth in peach roots by exogenous hydrogen sulfide based on RNA-Seq. Plant Physiol Biochem. 2021;159:179–92 33383385 10.1016/j.plaphy.2020.12.018

[24] Xi W , Feng J, Liu Y. et al. The R2R3-MYB transcription factor PaMYB10 is involved in anthocyanin biosynthesis in apricots and determines red blushed skin. BMC Plant Biol. 2019;19:287 31262258 10.1186/s12870-019-1898-4 PMC6604168

[25] Zhang Q , Feng C, Li W. et al. Transcriptional regulatory networks controlling taste and aroma quality of apricot (Prunus armeniaca L.) fruit during ripening. BMC Genomics. 2019;20:45 30646841 10.1186/s12864-019-5424-8 PMC6332858

[26] Verde I , Abbott AG, Scalabrin S. et al. The high-quality draft genome of peach (Prunus persica) identifies unique patterns of genetic diversity, domestication and genome evolution. Nat Genet. 2013;45:487–94 23525075 10.1038/ng.2586

[27] Verde I , Jenkins J, Dondini L. et al. The peach v2.0 release: high-resolution linkage mapping and deep resequencing improve chromosome-scale assembly and contiguity. BMC Genomics. 2017;18:225 28284188 10.1186/s12864-017-3606-9 PMC5346207

[28] Sayers EW , Bolton EE, Brister JR. et al. Database resources of the national center for biotechnology information. Nucleic Acids Res. 2022;50:D20–6 34850941 10.1093/nar/gkab1112 PMC8728269

[29] Cheng C , Liu J, Wang X. et al. PpERF/ABR1 functions as an activator to regulate PpPG expression resulting in fruit softening during storage in peach (Prunus persica). Postharvest Biol Technol. 2022;189:111919

[30] Gu C , Wang L, Wang W. et al. Copy number variation of a gene cluster encoding endopolygalacturonase mediates flesh texture and stone adhesion in peach. J Exp Bot. 2016;67:1993–2005 26850878 10.1093/jxb/erw021 PMC4783375

[31] Jiang L , Kang R, Feng L. et al. iTRAQ-based quantitative proteomic analysis of peach fruit (Prunus persica L.) at different ripening and postharvest storage stages. Postharvest Biol Technol. 2020;164:111137

[32] Nakano R , Kawai T, Fukamatsu Y. et al. Postharvest properties of ultra-late maturing peach cultivars and their attributions to melting flesh (M) locus: re-evaluation of M locus in association with flesh texture. Front Plant Sci. 2020;11:1817 10.3389/fpls.2020.554158 PMC7725752 33324428

[33] Qian M , Xu Z, Zhang Z. et al. The downregulation of PpPG21 and PpPG22 influences peach fruit texture and softening. Planta. 2021;254:22–12 34218358 10.1007/s00425-021-03673-6

[34] Zhu Y , Zeng W, Wang X. et al. Characterization and transcript profiling of PME and PMEI gene families during peach fruit maturation. J Am Soc Hortic Sci. 2017;142:246–59

[35] Bretó MP , Cantín CM, Iglesias I. et al. Mapping a major gene for red skin color suppression (highlighter) in peach. Euphytica. 2017;213

[36] Cao K , Ding T, Mao D. et al. Transcriptome analysis reveals novel genes involved in anthocyanin biosynthesis in the flesh of peach. Plant Physiol Biochem. 2018;123:94–102 29227951 10.1016/j.plaphy.2017.12.005

[37] Cheng J , Wei G, Zhou H. et al. Unraveling the mechanism underlying the glycosylation and methylation of anthocyanins in peach. Plant Physiol. 2014;166:1044–58 25106821 10.1104/pp.114.246876 PMC4213075

[38] Liu X , Chen M, Wen B. et al. Transcriptome analysis of peach (Prunus persica) fruit skin and differential expression of related pigment genes. Sci Hortic. 2019a;250:271–7

[39] Rahim MA , Busatto N, Trainotti L. Regulation of anthocyanin biosynthesis in peach fruits. Planta. 2014;240:913–29 24827911 10.1007/s00425-014-2078-2

[40] Ravaglia D , Espley RV, Henry-Kirk RA. et al. Transcriptional regulation of flavonoid biosynthesis in nectarine (Prunus persica) by a set of R2R3 MYB transcription factors. BMC Plant Biol. 2013;13:68 23617716 10.1186/1471-2229-13-68 PMC3648406

[41] Tuan PA , Bai S, Yaegaki H. et al. The crucial role of PpMYB10.1 in anthocyanin accumulation in peach and relationships between its allelic type and skin color phenotype. BMC Plant Biol. 2015;15:280 26582106 10.1186/s12870-015-0664-5 PMC4652394

[42] Zhao Y , Dong W, Zhu Y. et al. PpGST1, an anthocyanin-related glutathione S-transferase gene, is essential for fruit coloration in peach. Plant Biotechnol J. 2020;18:1284–95 31693790 10.1111/pbi.13291 PMC7152611

[43] Zhou H , Lin-Wang K, Wang H. et al. Molecular genetics of blood-fleshed peach reveals activation of anthocyanin biosynthesis by NAC transcription factors. Plant J. 2015;82:105–21 25688923 10.1111/tpj.12792

[44] Trainotti L , Tadiello A, Casadoro G. The involvement of auxin in the ripening of climacteric fruits comes of age: the hormone plays a role of its own and has an intense interplay with ethylene in ripening peaches. J Exp Bot. 2007;58:3299–308 17925301 10.1093/jxb/erm178

[45] Soto A , Ruiz KB, Ziosi V. et al. Ethylene and auxin biosynthesis and signaling are impaired by methyl jasmonate leading to a transient slowing down of ripening in peach fruit. J Plant Physiol. 2012;169:1858–65 22884412 10.1016/j.jplph.2012.07.007

[46] Wei J , Wen X, Tang L. Effect of methyl jasmonic acid on peach fruit ripening progress. Sci Hortic. 2017;220:206–13

[47] Tonutti P , Bonghi C, Ruperti B. et al. Ethylene evolution and 1-aminocyclopropane-1-carboxylate oxidase gene expression during early development and ripening of peach fruit. J Am Soc Hortic Sci. 1997;122:642–7

[48] Tatsuki M , Nakajima N, Fujii H. et al. Increased levels of IAA are required for system 2 ethylene synthesis causing fruit softening in peach (Prunus persica L. Batsch). J Exp Bot. 2013;64:1049–59 23364941 10.1093/jxb/ers381 PMC3580816

[49] Wang X , Ding Y, Wang Y. et al. Genes involved in ethylene signal transduction in peach (Prunus persica) and their expression profiles during fruit maturation. Sci Hortic. 2017;224:306–16

[50] Pan L , Zeng W, Niu L. et al. PpYUC11, a strong candidate gene for the stony hard phenotype in peach (Prunus persica L. Batsch), participates in IAA biosynthesis during fruit ripening. J Exp Bot. 2015;66:7031–44 26307136 10.1093/jxb/erv400 PMC4765781

[51] Bonghi C , Manganaris GA. Systems biology approaches reveal new insights into the molecular mechanisms regulating flesh fruit quality. In: Omics Technologies: Tools for Food Science. Taylor Francis group, 2012,25

[52] Tanaka Y , Sasaki N, Ohmiya A. Biosynthesis of plant pigments: anthocyanins, betalains and carotenoids. Plant J. 2008;54:733–49 18476875 10.1111/j.1365-313X.2008.03447.x

[53] Ma Y , Ma X, Gao X. et al. Light induced regulation pathway of anthocyanin biosynthesis in plants. Molecular sciences. 2021;22:11116 34681776 10.3390/ijms222011116 PMC8538450

[54] Santin M , Simoni S, Vangelisti A. et al. Transcriptomic analysis on the Peel of UV-B-exposed peach fruit reveals an Upregulation of phenolic- and UVR8-related pathways. Plan Theory. 2023;12:1818 10.3390/plants12091818 PMC10180693 37176875

[55] Zhou Y , Guo D, Li J. et al. Coordinated regulation of anthocyanin biosynthesis through photorespiration and temperature in peach (Prunus persica f. atropurpurea). Tree Genetics and Genomes. 2013;9:265–78

[56] Zhu YC , Zhang B, Allan AC. et al. DNA demethylation is involved in the regulation of temperature-dependent anthocyanin accumulation in peach. Plant J. 2020;102:965–76 31923329 10.1111/tpj.14680

[57] Liu W , Lin L, Zhang Z. et al. Gene co-expression network analysis identifies trait-related modules in Arabidopsis thaliana. Planta. 2019b;249:1487–501 30701323 10.1007/s00425-019-03102-9

[58] Lu Z , Cao H, Pan L. et al. Two loss-of-function alleles of the glutathione S-transferase (GST) gene cause anthocyanin deficiency in flower and fruit skin of peach (Prunus persica). Plant J. 2021;107:1320–31 33964100 10.1111/tpj.15312

[59] Zhao Y , Dong W, Wang K. et al. Differential sensitivity of fruit pigmentation to ultraviolet light between two peach cultivars. Front Plant Sci. 2017;8 10.3389/fpls.2017.01552 PMC5596067 28943881

[60] Wang DR , Yang K, Wang X. et al. Overexpression of MdZAT5, an C2H2-type zinc finger protein, regulates anthocyanin accumulation and salt stress response in apple Calli and Arabidopsis. Int J Mol Sci. 2022;23 10.3390/ijms23031897 PMC8836528 35163816

[61] Zhang L , Tao R, Wang S. et al. PpZAT5 suppresses the expression of a B-box gene PpBBX18 to inhibit anthocyanin biosynthesis in the fruit peel of red pear. Front Plant Sci. 2022;13:1–14 10.3389/fpls.2022.1022034 PMC9592862 36304405

[62] Dekker JP , Boekema EJ. Supramolecular organization of thylakoid membrane proteins in green plants. Biochim Biophys Acta Bioenerg. 2005;1706:12–39 10.1016/j.bbabio.2004.09.009 15620363

[63] Montané MH , Kloppstech K. The family of light-harvesting-related proteins (LHCs, ELIPs, HLIPs): was the harvesting of light their primary function? Gene. 2000;258:1–8 11111037 10.1016/s0378-1119(00)00413-3

[64] Leinonen R , Sugawara H, Shumway M. et al. The sequence read archive. Nucleic Acids Res. 2011;39:D19–21 21062823 10.1093/nar/gkq1019 PMC3013647

[65] Jung S , Lee T, Cheng C-H. et al. 15 years of GDR: new data and functionality in the genome database for Rosaceae. Nucleic Acids Res. 2019;47:D1137–45 30357347 10.1093/nar/gky1000 PMC6324069

[66] Ashburner M , Ball CA, Blake JA. et al. Gene ontology: tool for the unification of biology. Nat Genet. 2000;25:25–9 10802651 10.1038/75556 PMC3037419

[67] Carbon S , Douglass E, Good BM. et al. The gene ontology resource: enriching a GOld mine. Nucleic Acids Res. 2021;49:D325–34 33290552 10.1093/nar/gkaa1113 PMC7779012

[68] Mistry J , Chuguransky S, Williams L. et al. Pfam: the protein families database in 2021. Nucleic Acids Res. 2021;49:D412–9 33125078 10.1093/nar/gkaa913 PMC7779014

[69] Durinck S , Spellman PT, Birney E. et al. Mapping identifiers for the integration of genomic datasets with the R/bioconductor package biomaRt. Nat Protoc. 2009;4:1184–91 19617889 10.1038/nprot.2009.97 PMC3159387

[70] Kanehisa M , Goto S. KEGG: Kyoto encyclopedia of genes and genomes. Nucleic Acids Res. 2000;28:27–30 10592173 10.1093/nar/28.1.27 PMC102409

[71] Mi H , Ebert D, Muruganujan A. et al. PANTHER version 16: a revised family classification, tree-based classification tool, enhancer regions and extensive API. Nucleic Acids Res. 2021;49:D394–403 33290554 10.1093/nar/gkaa1106 PMC7778891

[72] Thimm O , Bläsing O, Gibon Y. et al. Mapman: a user-driven tool to display genomics data sets onto diagrams of metabolic pathways and other biological processes. Plant J. 2004;37:914–39 14996223 10.1111/j.1365-313x.2004.02016.x

[73] Kim D , Langmead B, Salzberg SL. HISAT: a fast spliced aligner with low memory requirements. Nat Methods. 2015;12:357–60 25751142 10.1038/nmeth.3317 PMC4655817

[74] Danecek P , Bonfield JK, Liddle J. et al. Twelve years of SAMtools and BCFtools. GigaScience. 2021;10:1–4 10.1093/gigascience/giab008 PMC7931819 33590861

[75] Li H , Handsaker B, Wysoker A. et al. The sequence alignment/map format and SAMtools. Bioinformatics. 2009;25:2078–9 19505943 10.1093/bioinformatics/btp352 PMC2723002

[76] Liao Y , Smyth GK, Shi W. featureCounts: an efficient general purpose program for assigning sequence reads to genomic features. Bioinformatics. 2014;30:923–30 24227677 10.1093/bioinformatics/btt656

[77] Wang Z , Gerstein M, Snyder M. RNA-Seq: a revolutionary tool for transcriptomics. Nat Rev Genet. 2009;10:57–63 19015660 10.1038/nrg2484 PMC2949280

[78] Ballouz S , Weber M, Pavlidis P. et al. EGAD: ultra-fast functional analysis of gene networks. Bioinformatics. 2017;33:612–4 27993773 10.1093/bioinformatics/btw695 PMC6041978

