# Supplementary figures and images for: Exploring large-scale gene coexpression networks in peach (Prunus persica L.): a new tool for predicting gene function
Source: Hortic Res. 2024 Jan 2;11(2):uhad294. doi: 10.1093/hr/uhad294 (PMC10939413; doi:10.1093/hr/uhad294)

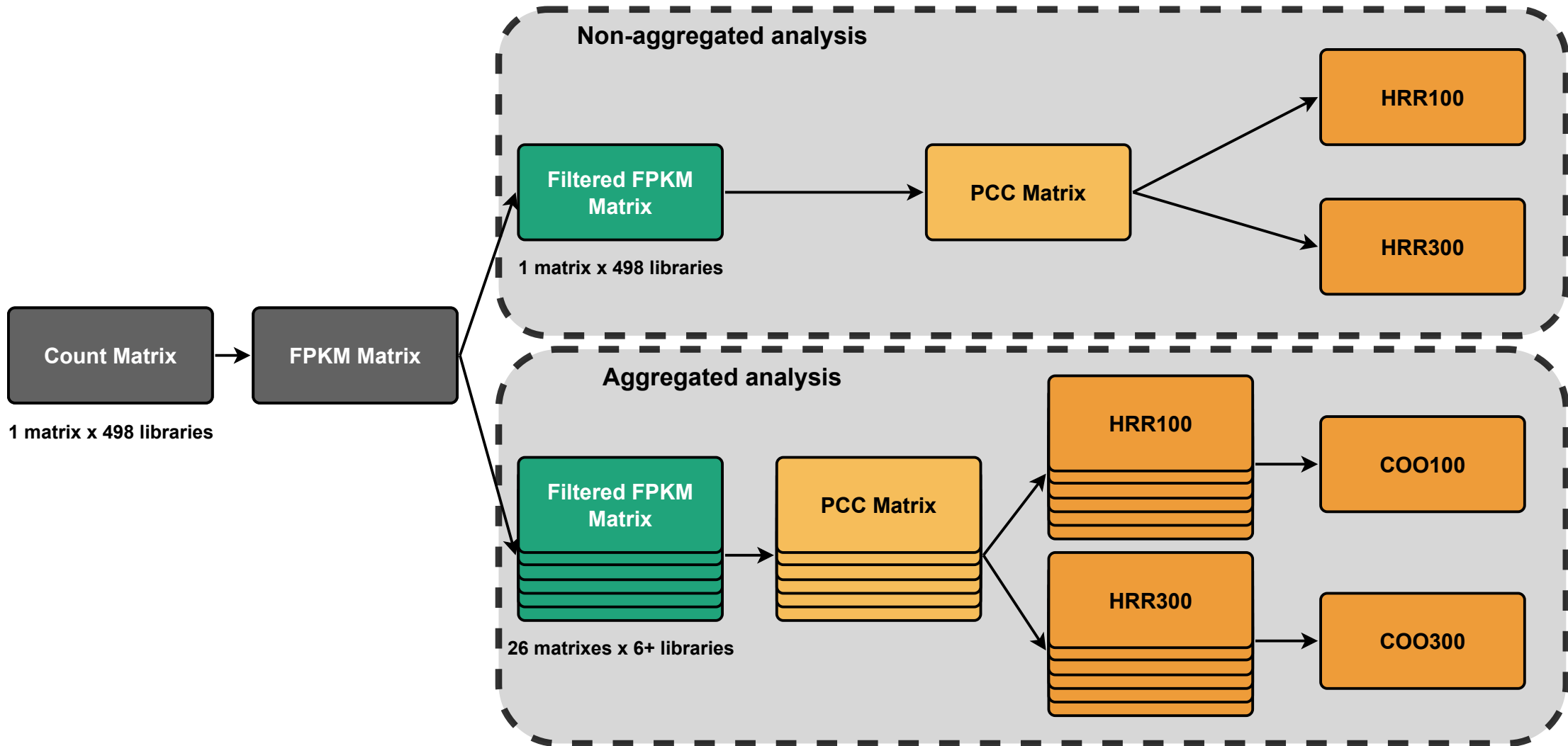

Supplement: Web_Material_uhad294 [file web_material_uhad294.zip › Supplementary Figure 1.pdf]
